# Associations between adrenal gland volume and adipose tissue compartments – a whole body MRI study

**DOI:** 10.1186/s12986-024-00823-x

**Published:** 2024-07-09

**Authors:** Esther Askani, Susanne Rospleszcz, Roberto Lorbeer, Charlotte Wintergerst, Katharina Müller-Peltzer, Lena S. Kiefer, Elias Kellner, Marco Reisert, Wolfgang Rathmann, Annette Peters, Christopher L. Schlett, Fabian Bamberg, Corinna Storz

**Affiliations:** 1 Department of Diagnostic and Interventional Radiology, Medical Center ‐ University of Freiburg, Faculty of Medicine, University of Freiburg, Freiburg, Germany; 2 Department of Epidemiology, Institute for Medical Information Processing, Biometry, and Epidemiology, Ludwig-Maximilians-University Munich, Munich, Germany; 3 Institute of Epidemiology, Helmholtz Center Munich, German Research Center for Environmental Health, Neuherberg, Germany; 4 Department of Radiology, Ludwig-Maximilans-University Hospital, Munich, Germany; 5 Department of Diagnostic and Interventional Radiology, Eberhard Karls University of Tuebingen, Tuebingen, Germany; 6 Medical Physics, Department of Radiology, Medical Center - University of Freiburg, Freiburg, Germany; 7 Institute of Biometrics and Epidemiology, German Diabetes Center, Duesseldorf, Germany; 8 German Center for Diabetes Research (DZD), Partner Site Neuherberg, Neuherberg, Germany; 9 German Center for Cardiovascular Disease Research (DZHK E.V.), Munich, Germany; 10 Department of Neuroradiology, Medical Center – University of Freiburg, Faculty of Medicine, University of Freiburg, Breisacher Str. 64, Freiburg, 79106 Germany

**Keywords:** Adrenal gland volume, Hypothalamic–pituitary–adrenal axis, MRI, Adipose tissue compartment

## Abstract

**Background:**

Obesity is associated with alterations in the hypothalamic–pituitary–adrenal (HPA) axis. Effects of glucocorticoids on adipose tissues appear to depend on the specific adipose depot, in which they take place. In this study, we aimed to investigate the role of MRI-based adrenal gland volume as an imaging marker in association with different adipose tissue compartments.

**Methods:**

The study cohort derives from the population-based research platform KORA (*Cooperative Health Research in the Augsburg Region, Germany)* MRI sub-study, a cross-sectional sub-study investigating the interactions between subclinical metabolic changes and cardiovascular disease in a study sample of 400 participants. Originally, eligible subjects underwent a whole-body MRI. MRI-based segmentations were performed manually and semi-automatically for adrenal gland volume, visceral adipose tissue (VAT), subcutaneous adipose tissue (SAT), epi- and pericardial fat and renal sinus fat. Hepatic and pancreatic lipid content were measured as pancreatic proton density fraction (PDFF) and MR-spectroscopic hepatic fat fraction (HFF). Multivariable linear regression analyses were performed.

**Results:**

A number of 307 participants (56.2 ± 9.1 years, 60.3% male, 14.3% with type 2 diabetes (T2DM), 30.6% with obesity, 34.2% with hypertension) were included. In multivariable analyses, strong positive associations between adrenal gland volume and VAT, total adipose tissue (TAT) as well as HFF persisted after extensive step-wise adjustment for possible metabolic confounders (VAT: beta = 0.31, 95%-CI [0.71, 0.81], *p* < 0.001; TAT: beta = 0.14, 95%-CI [0.06, 0.23], *p* < 0.001; HFF: beta = 1.17, 95%-CI [1.04, 1.31], *p* = 0.009). In contrast, associations between adrenal gland volume and SAT were attenuated in multivariate analysis after adjusting for BMI. Associations between pancreatic PDFF, epi- and pericardial fat and renal sinus fat were mediated to a great extent by VAT (pancreatic PDFF: 72%, epicardial adipose tissue: 100%, pericardial adipose tissue: 100%, renal sinus fat: 81.5%).

**Conclusion:**

Our results found MRI-based adrenal gland volume as a possible imaging biomarker of unfavorable adipose tissue distribution, irrespective of metabolic risk factors. Thus, adrenal gland volume may serve as a potential MRI-based biomarker of metabolic changes and contributes to an individual characterization of metabolic states and individual risk stratification. Future studies should elucidate in a longitudinal study design, if and how HPA axis activation may trigger unfavorable adipose tissue distribution and whether and to which extent this is involved in the pathogenesis of manifest metabolic syndrome.

**Supplementary Information:**

The online version contains supplementary material available at 10.1186/s12986-024-00823-x.

## Key points

° Effects of glucocorticoids on adipose tissues appear to depend on the specific adipose depot, in which they take place.

° MRI-based adrenal gland volume is a marker of unfavorable adipose tissue distribution.

° Visceral adipose tissue seems to have a predominant role in the interactions between adipose tissue depots and hypothalamic-pituitary-adrenal axis activation and consequently possibly also in the interactions occurring as part of the metabolic syndrome.

**Supplementary Information:**

The online version contains supplementary material available at 10.1186/s12986-024-00823-x.

## Introduction

Activation of the hypothalamic–pituitary–adrenal (HPA) axis resulting in excess of cortisol is observed in the context of physiological stress [1, 2]. The metabolic syndrome and obesity, which constitute a stressful metabolic condition, are associated with alterations in the HPA axis [3, 4]. However, whether HPA axis activation promotes the development of obesity and related comorbidities or represents an adaptive state to obesity is not yet understood [5]. On one side, adipose tissues produce mediators such as leptin that stimulate the HPA axis and may therefore cause adrenal hypertrophy [6], on the other side glucocorticoids, especially cortisol, also known as ‘stress hormone’ and produced in the adrenal gland, have effects on adipose tissue development and metabolism [7]. Moreover, the differentiated role and conduct of adipose tissue compartments in the context of obesity and pathogenesis of the metabolic syndrome is being observed [8, 9]. For instance, glucocorticoids seem to have a predominantly lipogenic effect in visceral adipose tissue (VAT) and a predominantly lipolytic role in subcutaneous adipose tissue (SAT) [10]. Coherently, previous data have shown a positive correlation between VAT and adrenal gland volume [4, 11]. Furthermore, previous studies have determined an association of different metabolic risk with different fat compartments. As VAT seems to be associated with a higher production of inflammatory cytokines, which lead to an increased metabolic activity, and a higher secretion of humeral mediators such as adiponectin and leptin, it has been linked to a higher mortality than SAT [12].

Only a few studies have used magnetic resonance imaging (MRI) for segmentation of adrenal gland volume, but due to the lack of radiation exposure MRI-based segmentation has a major advantage over computed tomography-(CT-)based segmentation [11, 13–16]. Comprehensive whole-body MR-imaging may serve as a screening tool for risk stratification in the future. Therefore, the evaluation of MRI-based imaging biomarkers is an important step towards the concept of a patient-adapted preventive medical setting.

Our previous research on MRI-based adrenal gland volume and impaired glucose metabolism has shown a significant correlation between type 2 diabetes (T2DM) as well as elevated triglyceride levels and increased adrenal gland volume, independently of BMI. However, in contrast to T2DM, the association of prediabetes and adrenal gland volume was confounded by BMI [17]. Furthermore, it was hypothesized that hyperactivity of the HPA axis may play a central role in the pathogenesis of both abdominal obesity and insulin resistance [18–20].

Therefore, in this study we aimed to assess associations between the volume of the adrenal glands and different adipose tissue compartments and to investigate the role of MRI-based adrenal gland volume as an imaging marker in this context.

Our hypothesis was that adrenal gland enlargement is correlated with unfavorable adipose tissue distribution, particularly with VAT and liver steatosis.

Furthermore, we aimed to investigate whether minor adipose tissues such as the pancreas, epi- and pericardial fat as well as renal sinus fat are associated with adrenal gland enlargement.

## Methods

### Study design and study population

The KORA (*Cooperative Health Research in the Augsburg Region, Germany*) MR-imaging study is a cross-sectional sub-study investigating the interactions between subclinical metabolic and cardiovascular disease in a study sample of 400 participants with prediabetes, T2DM, and normoglycemia from southern Germany. This KORA-MRI study is a subsample of the KORA FF4 Study (*n* = 2279), which is the second follow-up of the original population-based KORA S4 survey. Originally, inclusion criteria were: willingness to undergo whole-body MRI and qualification for being in the normoglycemic, prediabetes or T2DM group. Exclusion criteria were: age > 72 years, known prior cardiovascular disease, contraindications to contrast-enhanced MRI scan. For the present study, further exclusion criteria were a systemic cortisol intake, the detection of adrenal gland incidentalomas, poor image quality for the assessment of adrenal gland volume and missing data on adipose tissue compartments.

The KORA-MRI sub-study was approved by the Institutional Research Ethics Board of the Medical Faculty of Ludwig-Maximilian University, Munich. The requirements of the Helsinki declaration on human research were met. Prior to the MRI exams informed written consent was collected from each participant [21, 22].

### Whole-body MR-Imaging

Comprehensive whole-body MR-imaging was performed between June 2013 and September 2014 using a 3 Tesla Magnetom Skyra MRI (Siemens Healthcare, Erlangen, Germany) with a 18-channel body surface coil and a table-mounted spine matrix coil. The MR-protocol included sequences of the entire body (from neck to below hip). All images were analyzed by independent readers blinded to clinical characteristics of study participants [21].

### MR-Image analysis of adrenal gland volume

**Fig. 1:**
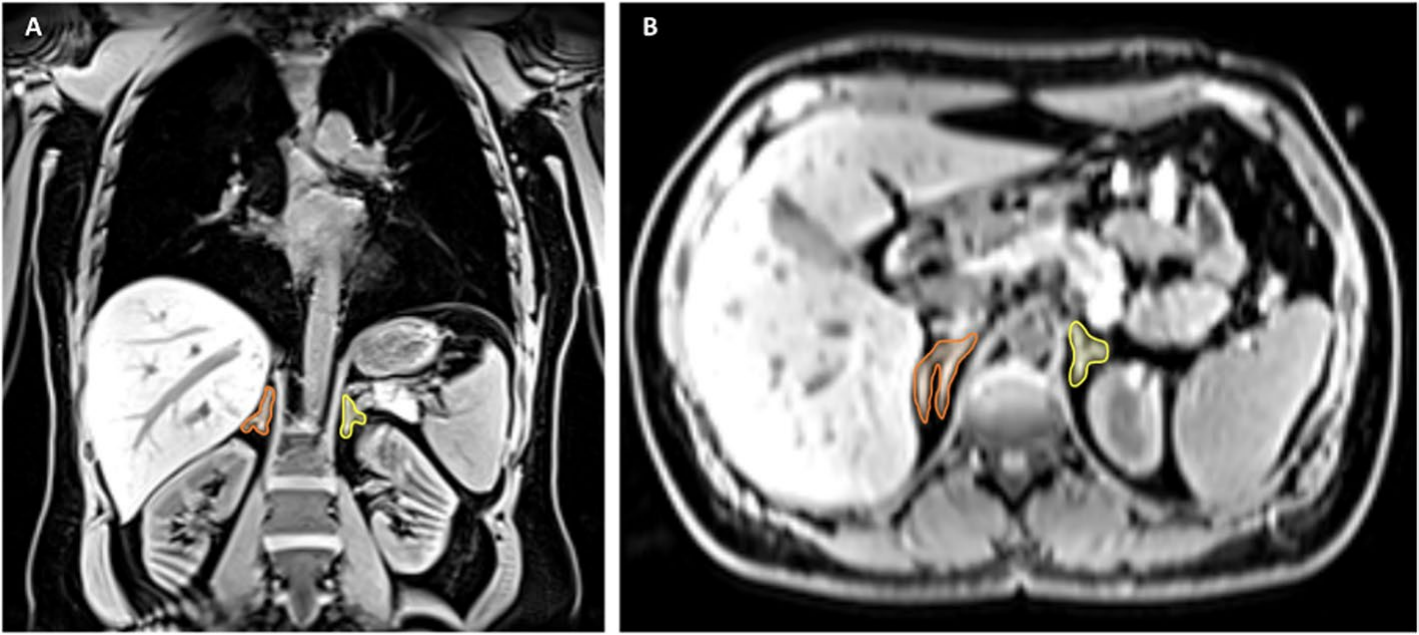
Example of adrenal gland segmentation of the right adrenal gland (orange) and of the left adrenal gland (yellow) in coronal (**A**) and axial (**B**) reconstructions on T1w-VIBE-Dixon sequences

For adrenal gland volume analysis, a two-point T1-weighted isotropic VIBE-Dixon gradient-echo sequence of the trunk was acquired with the following image parameters: slice thickness 1.7 mm, spatial resolution 1.7 × 1.7 mm^2^, field of view (FOV) 488 × 716 mm using a 256 × 256 matrix, repetition time (TR) 4.06 ms, echo time (TE) 1.26 & 2.49 ms, flip angle 9°. Adrenal glands were manually segmented on both sides on the water-only sequences of the two-point T1-weighted isotropic VIBE-Dixon gradient-echo sequence in axial, sagittal and coronal planes using the medical imaging platform NORA (www.nora.imaging.com). Adrenal gland volume was calculated automatically as the number of voxels multiplied by voxel size [17]. Figure 1 provides an example of adrenal gland segmentation in coronal and axial reconstructions.

### MR-Image analysis of VAT and SAT

**Fig. 2:**
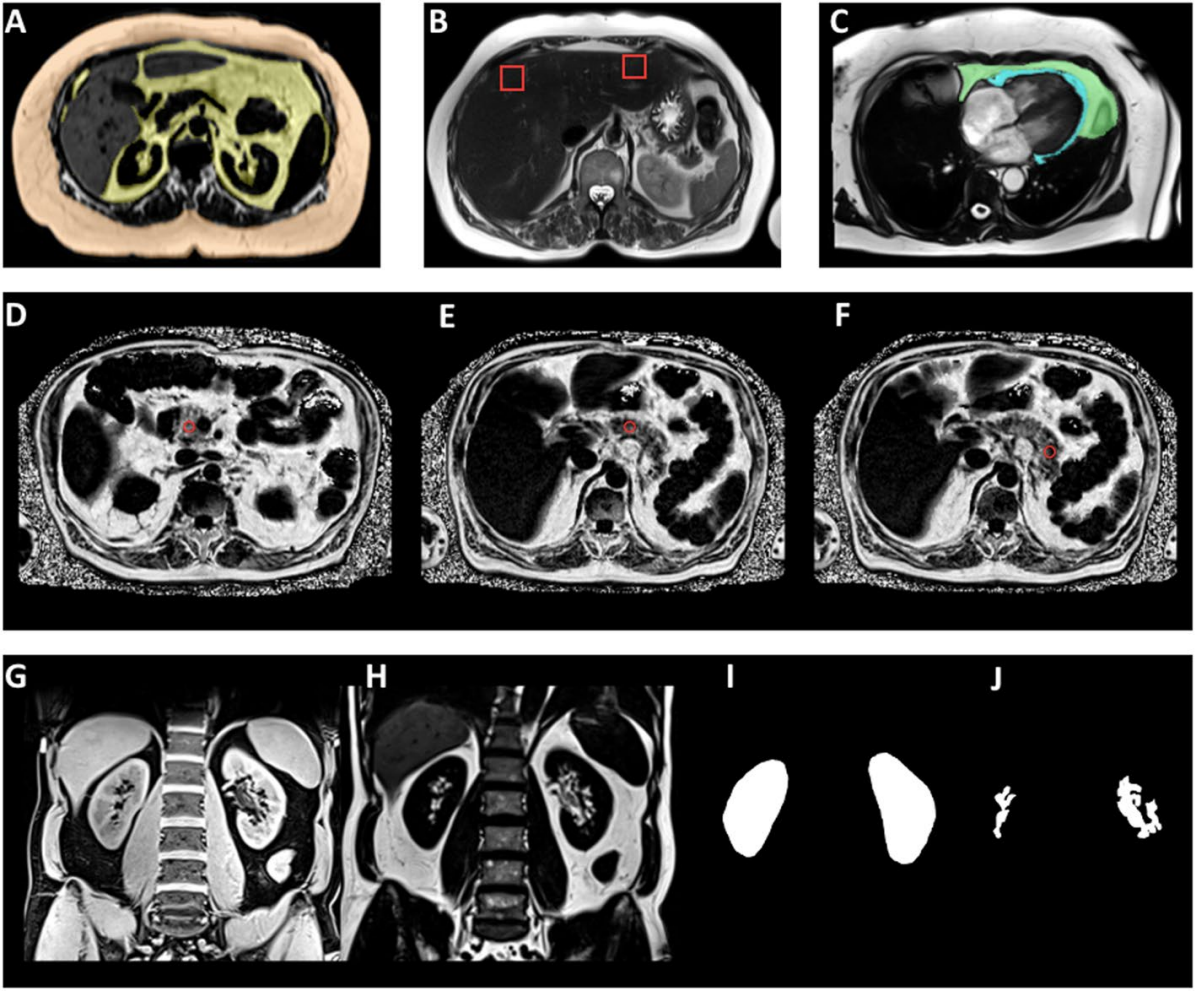
**A** Example of MRI-based VAT (yellow) and SAT (orange) assessment in axial reconstruction on a T1w-VIBE-Dixon (fat only) sequence. **B** Example of voxel placement on a T2 HASTE sequence in liver segment II and VIII on axial reconstruction for HFF quantification through spectroscopy. **C** Example of epi- (blue) and pericardial (green) fat compartment delineation on a steady-state free precession (SSFP) cine sequence. **D**-**F** Example of ROI placement in pancreatic head (**D**), body (**E**) and tail (**F**) for pancreatic PDFF measurement. **G**-**J** Example of renal sinus fat segmentation on a coronal T1w-VIBE-Dixon dataset (**G**) with corresponding fat only image (**H**) through generation of a whole kidney mask (**I**) and subsequent separation of renal sinus fat through thresholding the maximum pixel’s intensity (**J**)

For assessment of the adipose tissue compartments VAT and SAT a three-dimensional in/opposed VIBE-Dixon gradient-echo-sequence of the trunk was acquired with the following image parameters: slice thickness 1.7 mm, spatial resolution 1.7 × 1.7 mm^2^, FOV 488 × 716 mm using a 256 × 256 matrix, TR 4.06 ms, TE 1.26 & 2.49 ms, flip angle 9°. Quantification of VAT and SAT was achieved on a reconstructed fat selective tomogram by semi-automatical assessment with an in-house algorithm based on Matlab R2013a [23]. The amount of VAT was investigated from the femoral head to the cardiac apex. The amount of SAT was investigated from the femoral head to the diaphragm. Total adipose tissue (TAT) was obtained by the sum of VAT and SAT [21]. Figure 2A provides an example of MRI-based VAT and SAT assessment.

### MR-Image analysis of hepatic fat content

For assessment of liver fat content a modified single-voxel spectroscopy sequence with stimulated-echo acquisition mode (STEAM), implementing the high-speed T2-corrected multi-echo (HISTO) technique was used for 1 H Magnetic Resonance Spectroscopy (MRS), with the following image parameters: TR 3000 ms, mixing time between second and third radiofrequency pulses 10 ms, and five TEs (TE1 12 ms, TE2 24 ms, TE3 36 ms, TE4 48 ms, TE5 72 ms). A total of 1024 points were acquired at a bandwidth of 1200 Hz, with one signal acquired by using a voxel size of 30 × 30 × 30 mm^3^. Images were obtained during a breath-hold of 15 s. Voxels were placed in segment VIII of the right liver lobe and in segment II of the left liver lobe. Spectrum post-processing and lipid content estimation were automatically performed by a dedicated software package [24–26]. Mean liver fat signal fraction (HFF) was calculated from the measurement in the right (measured in segment VIII) and left (measured in segment II) liver lobe. Figure 2B provides an example of voxel placement in liver segment II and VIII.

### MR-Image analysis of pancreatic fat content

For assessment of pancreatic lipid content a six-point T1-weighted VIBE-Dixon sequence of the upper abdomen was acquired with the following image parameters: slice thickness 4 mm, FOV (read) 420 mm, FOV (phase) 78.1%, TR 8.9 ms, TE1 1.23 ms (opposed-phase), TE2 2.46 ms (in-phase), TE3 3.69 ms (opposed-phase), TE4 4.92 ms (in-phase), TE5 6.15 ms (opposed-phase), TE6 7.38 ms (in-phase), flip angle 4°. Images were obtained during a breath-hold of 15 s. Measurements account for confounders such as T2* decay, T1 bias, noise bias and fat composition. PDFF_pancreas_ was measured with the offline workstation syngo.via (Syngo Via, Siemens Healthcare, Erlangen, Germany). Regions of interest (ROIs) of approximately 100 mm^2^ were drawn into the pancreatic head, body and tail in different MRI-slices and the average PDFF was calculated out of these three ROIs [27]. Figure 2D-F provides an example of ROI placement in pancreatic head, body and tail.

### MR-Image analysis of epi- and pericardial fat

For assessment of epi- and pericardial fat a steady-state free precession (SSFP) cine sequence of the heart was acquired in the long axis 4-chamber view with the following image parameters: slice thickness 8 mm, FOV 297 × 360 mm using a 240 × 160 matrix, TR 29.97 ms, TE 1.46 ms, flip angle 63°. The epi- and pericardial fat compartment were manually delineated in the maximal systole with an openly available software (OsiriX Lite, Pixmeo, Bernex, Switzerland) [28]. Figure 2C provides an example of epi- and pericardial fat compartment delineation.

### MR-Image analysis of renal sinus fat

For assessment of renal sinus fat a coronal T1-weighted dual-echo Dixon and a coronal T2-weighted single shot fast spin echo (SS-FSE/HASTE) sequence were acquired. For the T1-weighted dual-echo Dixon sequence following parameters were applied: slice thickness 1.7 mm, FOV 488 × 716 mm using a 256 × 256 matrix, TR 4.06 ms, TE 1.26 & 2.49 ms, flip angle 9°. For the T2 HASTE sequence following image parameters were applied: slice thickness 5 mm, FOV 296 × 380 mm using a 320 × 200 matrix, TR 1000 ms, TE 91 ms, flip angle 131°. Renal sinus fat was assessed semi-automatically using Matlab (Version R2011b, The MathWorks, Natick, USA) [29]. Kidney segmentation was achieved by thresholding the T1w Dixon images, generating an entire kidney mask. Within this kidney mask, renal parenchyma and renal sinus and sinus fat were determined by thresholding the maximum pixel’s intensity and the renal sinus fat was subsequently separated. The final volume was calculated by voxel summation [30]. Figure 2G-J provides an example of renal sinus fat evaluation.

### Other covariates

Health assessment for the collection of demographic and clinical data was obtained in a uniform fashion through standardized interviews, laboratory and physical exams at the KORA study center between 2013 and 2014 [21, 22, 31]. The Framingham risk score for 10 year CVD risk was calculated according to the published formula with recalibration to mean risk factor values for the sample at hand [32].

### Statistical analysis

Demographic, lifestyle and metabolic risk factors as well as MRI parameters relating to adrenal gland volume and adipose tissue compartments are described as arithmetic means with standard deviations (SDs) or medians with interquartile range for continuous covariates and counts and percentages for categorical covariates. Correlations of adrenal gland volume with risk factors and adipose tissue compartments were assessed by Spearman’s correlation coefficient with corresponding 95% confidence intervals (CI) for the whole sample as well as sex-stratified. Associations between exposure adrenal gland volume with outcomes adipose tissue compartments were calculated by linear regression models with exposure and outcomes standardized before analysis. Outcomes HFF, PDFF_pancreas_ and renal sinus fat were log-transformed to improve model fit. Estimates are given as beta coefficients (as increase in SD of outcome per SD of exposure) or percent change (per SD of exposure) of the geometric mean (of standardized outcome) with corresponding 95% CIs. Models were adjusted as follows: Model 1: age and sex, Model 2: age, sex and lifestyle factors (alcohol consumption, smoking, physical activity), Model 3: age, sex, lifestyle and metabolic risk factors (hypertension, T2DM, increased triglycerides), Model 4: age, sex, lifestyle, metabolic risk factors and BMI, Model 5: age, sex, lifestyle, metabolic risk factors and VAT. Furthermore, models were calculated stratified by sex; in this case, outcomes and exposure were also standardized by sex. Additionally, models were calculated separately for left and right adrenal gland. To explore the dependency of the association on anthropometric factors, BMI was exchanged with either waist circumference, waist-to-hip ratio, body surface area, VAT, or body weight in further models. To further characterize the role of VAT, causal mediation models with VAT as a mediating variable were calculated (R package ‘mediate’). Furthermore, subgroup analyses according to sex, weight status, diabetes status, hypertension, cardiovascular risk, lipid profile and physical activity were performed. *P*-values < 0.05 were considered to denote statistical significance. R version 4.1.2 was used for all analyses.

## Results

For the 400 participants originally included in the KORA MRI sub-study, availability of the required MRI sequences for adrenal gland volume and individual adipose tissue evaluation varied. MRI-based segmentation and analysis could be performed for adrenal gland volume in 375 participants, for VAT in 369 participants, for SAT in 367 participants, for TAT in 364 participants, for HFF in 369 participants, for PDFF_pancreas_ in 367 participants, for epi- and pericardial fat in 349 participants, and for renal sinus fat in 351 participants. Two participants were taking systemic cortisol medication. In two cases, adrenal gland incidentalomas were detected. Overall, only considering participants with overlapping datasets for the individual adipose tissue compartments and adrenal gland volume, and only considering participants without systemic cortisol intake or incidentalomas, 307 participants were eligible for analysis of associations between adrenal gland volume and adipose tissue compartments. Out of these 307 participants (56.2 ± 9.1 years, 60.3% male) 188 (61.2%) were classified as normoglycemic, 75 (24.4%) had prediabetes, and 44 (14.3%) had diabetes. Overweight was detected in 139 (45.3%) participants and obesity in 94 (30.6%) participants. In 105 (34.2%) participants hypertension was detected. Participant’s demographic, clinical and lipometabolic parameters are depicted in Table 1.

**Table 1:** Participant’s demographic, clinical and lipometabolic parameters

	**All**	**Men**	**Women**
N	307	185 (60.3%)	122 (39.7%)
**Demographic characteristics**			
Age	56.2 ± 9.1	56.2 ± 9.2	56.1 ± 9.0
**Anthropometric characteristics**			
Height (cm)	171.8 ± 9.8	177.6 ± 6.7	163.1 ± 6.8
BMI (kg/m^2^)	28.2 ± 4.6	28.5 ± 4.1	27.9 ± 5.2
Normal (< 25 kg/m^2^)	74 (24.1%)	34 (18.4%)	40 (32.8%)
Overweight (25–29,9 kg/m^2^)	139 (45.3%)	96 (51.9%)	43 (35.2%)
Obese (≥ 30 kg/m^2^)	94 (30.6%)	55 (29.7%)	39 (32.0%)
Waist circumference (cm)	99.0 ± 13.4	103.5 ± 11.6	92.2 ± 13.1
Waist-to-Hip Ratio	0.92 ± 0.09	0.97 ± 0.07	0.86 ± 0.07
Body Surface Area	2.0 ± 0.2	2.1 ± 0.2	1.8 ± 0.2
**Lifestyle factors**			
Alcohol consumption (g/d) (median [IQR])	10.3 [1.0, 27.5]	20.0 [3.7, 40.2]	2.9 [0.0, 13.0]
No (0 g/d)	71 (23.1%)	31 (16.8%)	40 (32.8%)
Moderate (0.1–39.9 g/d for men and 0.1–19.9 g/d for women)	175 (57.0%)	106 (57.3%)	69 (56.6%)
Heavy (≥ 40 g/d for men and ≥ 20 g/d for women)	61 (19.9%)	48 (25.9%)	13 (10.7%)
Smoking			
Never-smoker	111 (36.2%)	61 (33.0%)	50 (41.0%)
Ex-smoker	136 (44.3%)	89 (48.1%)	47 (38.5%)
Smoker	60 (19.5%)	35 (18.9%)	25 (20.5%)
Physically active (≥ 1h/week)	182 (59.3%)	106 (57.3%)	76 (62.3%)
**Metabolic risk factors**			
Triglycerides (mg/dl)	135.7 ± 89.9	156.0 ± 103.9	104.9 ± 49.3
Increased Triglycerides (> 150mg/dl)	90 (29.3%)	68 (36.8%)	22 (18.0%)
Total cholesterol (mg/dl)	218.6 ± 37.5	217.2 ± 39.1	220.8 ± 35.0
HDL (mg/dl)	60.7 ± 17.9	54.9 ± 15.2	69.6 ± 18.1
LDL (mg/dl)	141.0 ± 33.3	142.7 ± 34.2	138.6 ± 31.9
Hypertension	105 (34.2%)		
Systolic blood pressure (mmHg)	121.2 ± 16.7	126.5 ± 16.4	113.2 ± 13.8
Diastolic blood pressure (mmHg)	75.7 ± 10.0	77.8 ± 10.4	72.6 ± 8.5
Normoglycemia	188 (61.2%)	102 (55.1%)	86 (70.5%)
Prediabetes	75 (24.4%)	49 (26.5%)	26 (21.3%)
Diabetes	44 (14.3%)	34 (18.4%)	10 (8.2%)
HbA1c (%)	5.6 ± 0.8	5.6 ± 0.9	5.5 ± 0.5
Fasting glucose (mg/dL)	104.6 ± 23.9	108.1 ± 26.6	99.1 ± 17.6
2-h Glucose (mg/dL)	114.2 ± 42.7	119.0 ± 47.0	107.3 ± 34.5
hs-CRP mg/L (median[IQR])	1.2 [0.6, 2.5]	1.1 [0.6, 2.3]	1.4 [0.7, 2.8]
Framingham Risk Score, (%)	11.6 ± 10.9	14.9 ± 12.1	6.7 ± 6.0
**Adrenal Glands**			
Adrenal gland volume, ml (right)	5.3 ± 2.2	6.2 ± 2.1	3.9 ± 1.5
Adrenal gland volume, ml (left)	6.1 ± 2.5	7.1 ± 2.4	4.6 ± 1.7
Adrenal gland volume, ml (sum of right and left)	11.4 ± 4.3	13.3 ± 4.0	8.5 ± 2.9
Adrenal gland volume, ml (average of left and right)	5.7 ± 2.1	6.6 ± 2.0	4.2 ± 1.4
**Adipose tissue**			
TAT (l)	12.9 ± 5.3	13.3 ± 5.2	12.2 ± 5.3
VAT (l)	4.7 ± 2.6	5.8 ± 2.5	3.0 ± 1.8
SAT (l)	8.2 ± 3.6	7.5 ± 3.3	9.2 ± 3.9
HFF (%)	8.9 ± 7.8	10.5 ± 8.3	6.4 ± 6.3
Pancreatic PDFF (%)	7.8 ± 6.9	8.8 ± 7.8	6.3 ± 4.9
Renal sinus adipose tissue (%)	64.4 ± 9.8	67.6 ± 7.0	59.4 ± 11.2
Epicardial adipose tissue (cm^2^)	8.1 ± 4.2	9.4 ± 4.3	6.3 ± 3.2
Pericardial adipose tissue (cm^2^)	27.0 ± 15.3	33.4 ± 15.6	17.2 ± 8.0

Data are given as mean and standard deviation, or counts and percentages, unless indicated otherwise

*TAT* total adipose tissue, *VAT* visceral adipose tissue, *SAT* subcutaneous adipose tissue, *HFF* hepatic fat fraction, *PDFF* proton-density fat fraction, *IQR* interquartile range

A regression model on unstandardized outcome adrenal gland volume and exposure unstandardized body weight (adjusted for age, sex, lifestyle and metabolic risk factors) revealed an increase of 0.12 ml in adrenal gland volume per 1 kg body weight (beta = 0.12, 95%-CI [0.10, 0.15], *p* < 0.001).

### Adrenal gland volume and VAT, TAT & SAT

**Fig. 3:**
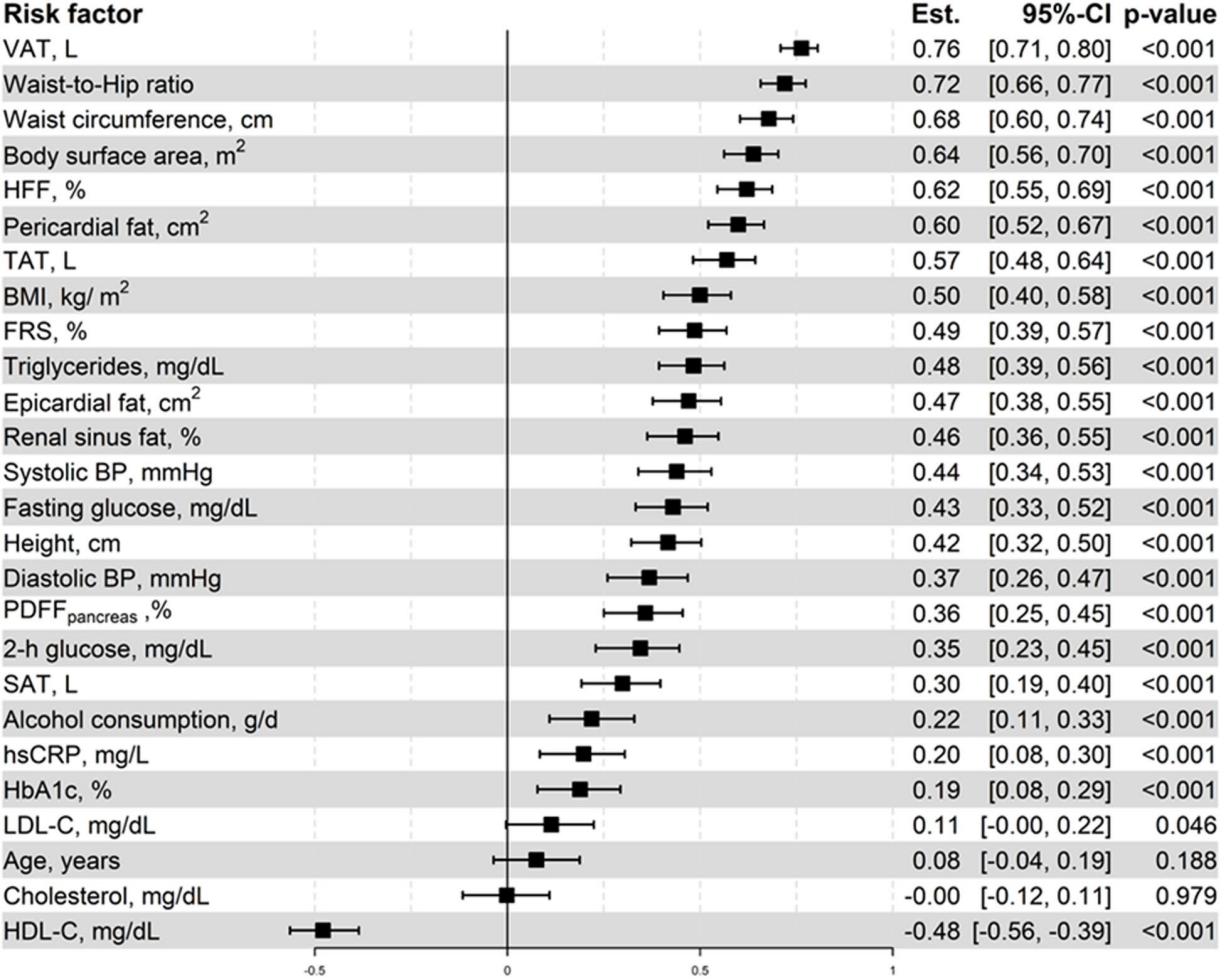
Correlation of adrenal gland volume with adipose tissue depots and metabolic risk factors. Estimate denotes Spearman’s correlation coefficient

**Fig. 4:**
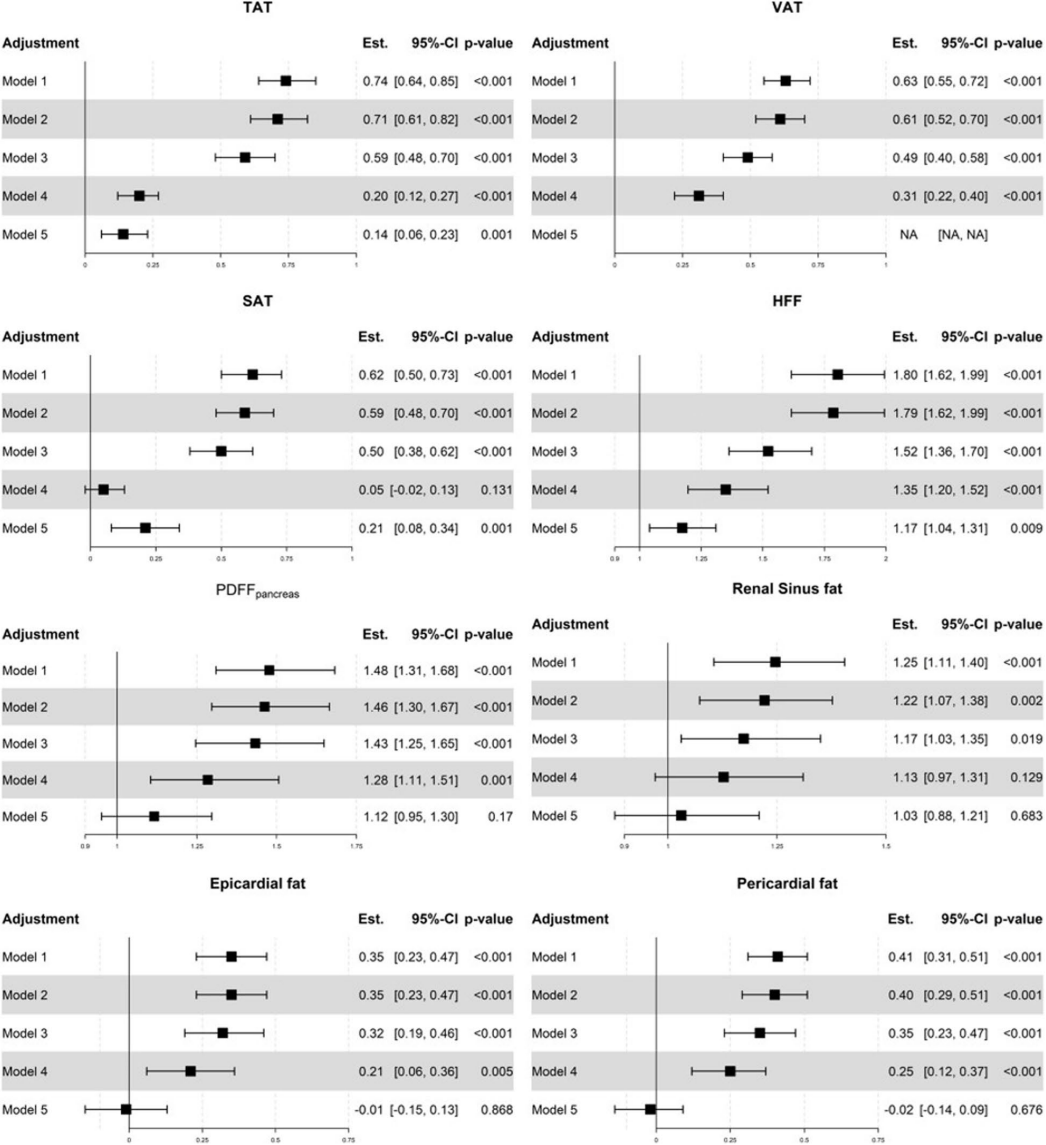
Associations of adrenal gland volume with adipose tissue compartments. Results from a linear regression model with outcome adipose tissue and exposure adrenal gland volume. Outcomes and exposure were standardized before analysis. Outcomes HFF, PDFF_pancreas_ and renal sinus fat were log-transformed before analysis and estimates represent percent change of the mean. For all other outcomes, estimates are given as beta coefficients. Adjustments: Model 1: age and sex, Model 2: age, sex and lifestyle factors (alcohol consumption, smoking, physical activity), Model 3: age, sex, lifestyle and metabolic risk factors (hypertension, diabetes, increased triglycerides), Model 4: age, sex, lifestyle, metabolic risk factors and BMI. Model 5: age, sex, lifestyle, metabolic risk factors and VAT

**Fig. 5:**
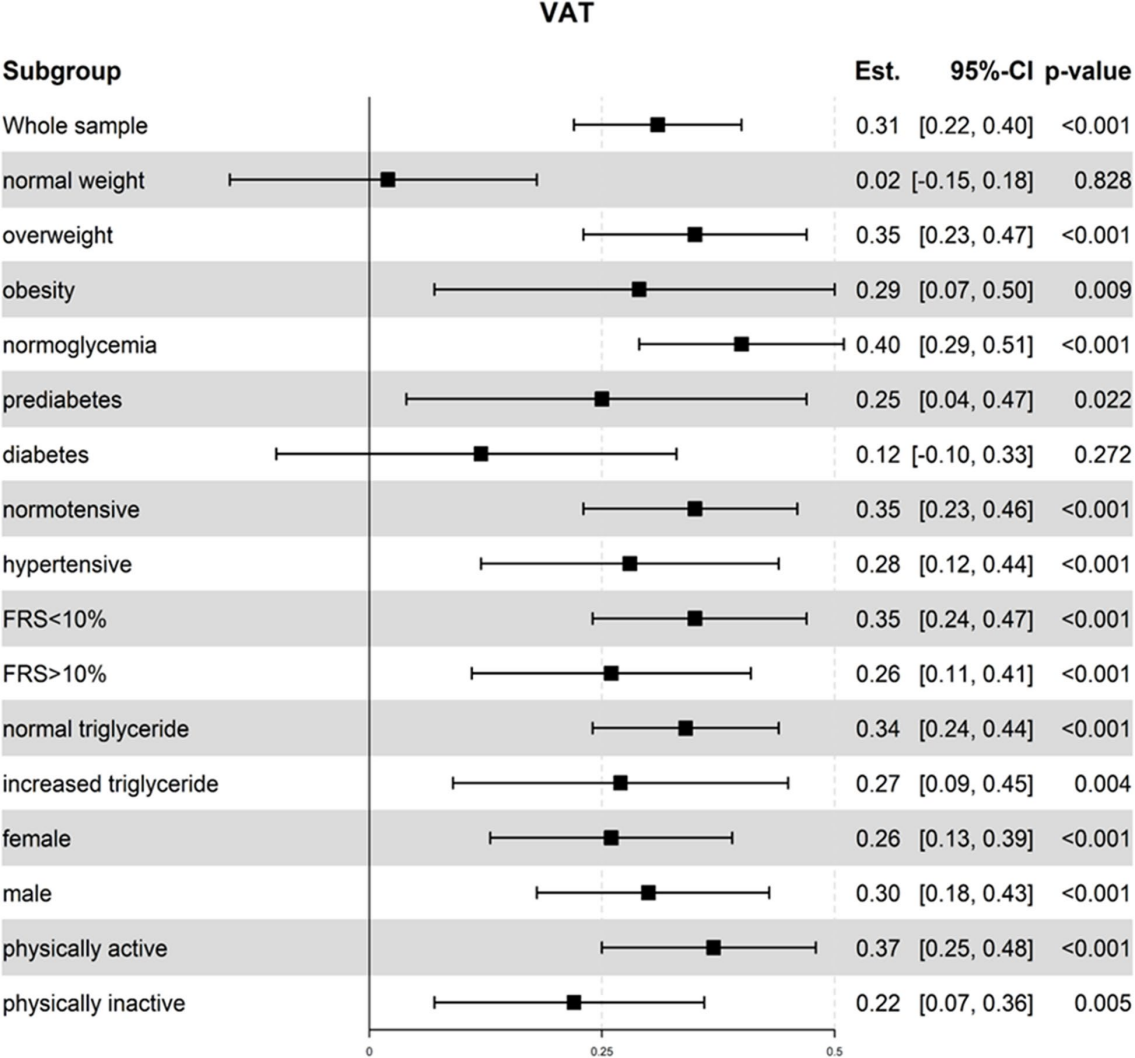
Association of adrenal gland volume with VAT in different subgroups. Results from a linear regression model with outcome VAT and exposure adrenal gland volume. Outcome and exposure were standardized before analysis. Estimates are given as beta

Univariate analysis showed that all adipose tissues were significantly associated with adrenal gland volume. VAT showed the strongest association (Spearman’s correlation coefficient = 0.76, 95%-CI [0.71, 0.81], *p* < 0.001) and SAT the weakest association with adrenal gland volume (Spearman’s correlation coefficient = 0.30, 95%-CI [0.20, 0.39], *p* < 0.001) (Fig. 3, Supplementary Fig. 1). In sex-stratified analyses, correlation of VAT with adrenal gland volume was equal in men and women (*r* = 0.65, 95%-CI [0.55, 0.73], and [0.54, 0.74] in men and women respectively, Supplementary Figs. 2 and 3). The association with adrenal gland volume persisted for VAT after adjusting for age, sex, lifestyle, metabolic risk factors and BMI (beta = 0.31, 95%-CI [0.71, 0.81], *p* < 0.001, Model 4) and for TAT after adjusting for age, sex, lifestyle, metabolic risk factors and BMI or VAT (beta = 0.20, 95%-CI [0.12, 0.27], *p* < 0.001, Model 4; beta = 0.14, 95%-CI [0.06, 0.23], *p* < 0.001, Model 5); for SAT, association with adrenal volume was attenuated after adjusting for BMI (beta = 0.05, 95%-CI [-0.02, 0.13], *p* = 0.131, Model 4), not however after adjusting for VAT (beta = 0.21, 95%-CI [0.08, 0.34], *p* = 0.001, Model 5) (Fig. 4). Results were similar when adjusting for other anthropometric measures (Supplementary Fig. 4). Interestingly, when analyzing different subgroups, participants with T2DM and participants with normal weight did not show a significant association between adrenal gland volume and VAT or TAT (Fig. 5, Supplementary Fig. 5). In sex-stratified analyses, results were comparable between men and women (Supplementary Figs. 6 and 7). However, associations of adrenal gland volume with TAT, VAT and SAT were slightly stronger in men, and the association with SAT persisted after adjustment for BMI (Supplementary Fig. 6). Results for left and right adrenal gland volume were comparable (Supplementary Table 1).

### Adrenal gland volume and HFF & pancreatic PDFF

HFF and pancreatic PDFF both showed significant associations with adrenal gland volume before adjustment for possible confounders (HFF: Spearman’s correlation coefficient = 0.62, 95%-CI [0.54, 0.69], *p* < 0.001; PDFF_pancreas_: Spearman’s correlation coefficient = 0.36, 95%-CI [0.25, 0.46], *p* < 0.001) (Fig. 3).

The association with adrenal volume persisted for HFF after adjusting for age, sex, lifestyle, metabolic risk factors and BMI or VAT (beta = 1.35, 95%-CI [1.20, 1.52], *p* < 0.001, Model 4; beta = 1.17, 95%-CI [1.04, 1.31], *p* = 0.009, Model 5); for PDFF_pancreas_, the association with adrenal gland volume was attenuated after adjustment for VAT (beta = 1.12, 95%-CI [0.95, 1.30], *p* = 0.17, Model 5) (Fig. 4). Mediation analysis showed that 72% of the association between adrenal gland volume and PDFF_pancreas_ were mediated by VAT. In sex-stratified analyses, the association of adrenal gland volume and HFF was stronger in women compared to men, and persisted after adjustment for BMI or VAT (Supplementary Fig. 7). On the other hand, the association of adrenal gland volume with PDFF_pancreas_ attenuated immediately in women after any adjustment besides age, whereas in men, the association remained statistically significant (Supplementary Fig. 6). Results for left and right adrenal gland volume were comparable (Supplementary Table 1).

### Adrenal gland volume and epi- and pericardial adipose tissue

Epi- and pericardial adipose tissue were both significantly associated with adrenal gland volume in univariate analysis (epicardial adipose tissue: Spearman’s correlation coefficient = 0.47, 95%-CI [0.38, 0.55], *p* < 0.001; pericardial adipose tissue: Spearman’s correlation coefficient = 0.60, 95%-CI [0.52, 0.67], *p* < 0.001) (Fig. 3). For both cardial adipose tissue departments the association with adrenal gland volume was attenuated after adjustment for VAT (epicardial adipose tissue: beta = -0.01, 95%-CI [-0.15, 0.13], *p* = 0.868, Model 5; pericardial adipose tissue: beta = -0.02, 95%-CI [-0.14, 0.13], *p* = 0.09, Model 5) (Fig. 4). Mediation analysis showed that in both cases 100% of the associations between adrenal gland volume and epicardial as well as pericardial adipose tissue were mediated by VAT, indicating lack of a direct effect. In sex-stratified analyses, results were comparable between men and women (Supplementary Figs. 6 and 7). However, associations of adrenal gland volume with epi- and pericardial adipose tissue were generally stronger in men, Results for left and right adrenal gland volume were comparable (Supplementary Table 1).

### Adrenal gland volume and renal sinus adipose tissue

Renal sinus adipose tissue was associated with adrenal gland volume in univariate analysis (Spearman’s correlation coefficient = 0.46, 95%-CI [0.36, 0.55], *p* < 0.001) (Fig. 3). However, this association was attenuated after adjustment for BMI (beta = 1.13, 95%-CI [0.97, 1.31], *p* = 0.129, Model 4) and after adjustment for VAT (beta = 1.03, 95%-CI [0.88, 1.31], *p* = 0.121, Model 5) (Fig. 4). Mediation analysis showed that 81.5% of the association between adrenal gland volume and renal sinus adipose tissue were mediated by VAT. In sex-stratified analysis, an association of adrenal gland volume with renal sinus adipose tissue was present in men, which remained after adjustment (Supplementary Fig. 6), and only attenuated after adjustment for VAT. Results for left and right adrenal gland volume were comparable (Supplementary Table 1).

## Discussion

The present study investigated associations between MRI-based adrenal gland volume and different adipose tissue compartments and found MRI-based adrenal gland volume as a marker of unfavorable adipose tissue distribution, irrespective of anthropometric measurements and largely independent of metabolic risk factors.

Our results showed strong correlations between adrenal gland volume and VAT, TAT and HFF, independent of possible confounders, while the association between adrenal gland volume and SAT revealed a dependency on BMI. Furthermore, the associations between adrenal gland volume and PDFF_pancreas_, epi- and pericardial adipose tissue, and renal sinus adipose tissue were revealed to be mediated by VAT to different degrees. Moreover, analysis of different subgroups showed that participants with T2DM and with normal weight did not reveal a correlation between adrenal gland volume and VAT, TAT or HFF. Overall, our results are the first comprehensive analysis of associations between MRI-based adrenal gland volume and various MRI-based adipose tissues and support the notion of complex adrenal-adipose interplays, in which adrenal gland volume seems to be associated with unfavorable adipose tissue distribution. As an imaging biomarker, adrenal gland volume could therefore serve as an indication of metabolic changes and thus contribute to individual risk stratification.

In comparison with the other investigated adipose tissue compartments, VAT revealed a predominant association with adrenal gland volume. Since we observed an association between VAT and increased adrenal gland volume independently of BMI, we assume that this correlation cannot solely be attributed to an increase in adipose tissue, but is rather due to a complex mechanism of metabolic dysregulation. This may reflect previous findings discussing different metabolic risk of different fat compartments [18, 33]. More specifically, Storz et al. found that among measurements, VAT was stronger related to prediabetes or diabetes as compared to TAT, BMI, or waist circumference, while the association of SAT was not independent of potential confounders [34]. Also, in a large sample drawn from the Framingham Heart Study, including participants without prior cardiovascular diseases, VAT was more strongly associated with adverse metabolic risk profile as compared to SAT [33]. The role of SAT in the development of the metabolic syndrome and in cardiometabolic risk has not yet been quite solved. While some studies indicate that SAT could have beneficial effects on metabolism [35], other findings have led to the assumption that SAT may contribute to the metabolic syndrome [36]. Our findings have adduced similarly conflicting results, revealing BMI as a confounder of the association between SAT and adrenal gland volume.

In addition, we found that TAT was associated to adrenal gland volume as well. Since TAT was obtained by the sum of VAT and SAT, it could be assumed that the correlation found between TAT and adrenal gland volume may be influenced by the preexisting strong correlation between VAT and adrenal gland volume. However, the correlation between TAT and adrenal gland volume persisted even after adjusting for VAT.

Obesity and the metabolic syndrome result in ectopic fat depositions also in other organ systems, including the liver, the pancreas, the heart, and the kidney. For example, it has been shown that increased hepatic fat quantitatively measured by MRI-PDFF had a significant dose-relationship with the presence of the metabolic syndrome in patients with nonalcoholic fatty liver disease [37]. In our study, correlations between HFF and adrenal gland volume persisted after extensive adjustment for metabolic confounders and no mediating effect of VAT was detected, suggesting direct interactions between the liver fat depot and activation of the HPA axis.

Associations between PDFF_pancreas_ and adrenal gland volume were revealed to be mediated for a great part by VAT. This may partly be explained methodologically, since due to the lobulation of the pancreas VAT could have confounded PDFF measurements.

In recent years, the epicardial and the pericardial adipose tissue have attracted much attention and the relationship between these adipose tissue compartments and heart disease has been investigated [38]. Additionally, it was also shown that epi- and pericardial adipose tissue are associated not only with cardiovascular disease but also with the metabolic syndrome [39]. We found, that an initially observed relationship between adrenal gland volume and epicardial as well as pericardial adipose tissue was mediated by VAT. This is in line with previous findings that suggested pericardial fat as an overall marker of visceral fat accumulation [40].

Ectopic fat accumulation in the renal sinus has been shown in previous studies to be associated with increased risk of hypertension and chronic renal disease [41]. This is explained by the space-occupying effect of fat accumulation in the renal sinus resulting in increased hydrostatic pressure through compression of renal veins, ultimately activating the renin angiotensin aldosterone system and consequently stimulating the adrenal gland [42]. However, in our study associations between renal sinus adipose tissue and adrenal gland volume were mediated to a large extent by VAT.

Altogether, our observations on ectopic fat compartments emphasize the predominant role of VAT in the interactions between adipose tissue depots and HPA axis activation and consequently possibly also in the interactions occurring as part of the metabolic syndrome.

Future studies should elucidate in a longitudinal study design, if and how HPA axis activation may trigger unfavorable adipose tissue distribution and whether these may develop into manifest metabolic syndrome. Further understanding of a possible HPA axis involvement in the development of the metabolic syndrome may lead to early MRI-based detection and intervention strategies targeting HPA axis activation.

Interestingly, analysis of different subgroups showed that participants with T2DM did not reveal a correlation between adrenal gland volume and VAT or TAT. This may be explained by the already strong associations existing between adrenal gland volume and T2DM [17]. However, we don’t have a clear explanation for the missing correlation in this subgroup. Further studies are necessary to establish to which extent for example therapeutic interventions for T2DM such as medication or changes in lifestyle may be responsible for adrenal gland volume decrease.

Also, the normal weight subgroup revealed a missing correlation between the subgroup and VAT or TAT. This observation may be due to the rather small variances in adrenal gland volume as well as in VAT in this subgroup through which small effects may not reveal themselves statistically.

Some limitations of our study warrant mentioning. The cross-sectional design of our study does not permit final conclusions on the order of events. Thus, our results do not allow resolutions on the cause or the development of adipose tissue depots and HPA axis activation. The European and asymptomatic population from the region of Augsburg decreases the generalizability of our results to other ethnicities or geographical regions. Furthermore, no systemic recording of cortisol laboratory parameters was carried out, so that we were unable to correlate the adrenal gland volume with corresponding laboratory parameters. Knowledge of cortisol levels would have strengthened our observations on HPA axis activation. Moreover, data on Cushing’s syndrome, primary aldosteronism, and pheochromocytoma were missing, however prevalence of these diseases is low, so that we do not expect significant alteration of the results through the potential existence of these diseases in the study cohort. Furthermore, MRI-based adrenal gland volume is a rather new biomarker for chronic HPA axis activation, but the feasibility of MRI-based adrenal gland segmentation has been shown [17], and ACTH which mainly stimulates the production and release of cortisol is considered the predominant if not the exclusive trophic factor for the adrenals [43].

Future studies with longitudinal designs and a systemic recording of cortisol laboratory parameters are necessary to gain an understanding of the complex interplays regarding the development of the metabolic syndrome.

## Conclusion

In this study, we performed a comprehensive analysis of associations between MRI-based adrenal gland volume and various MRI-based adipose tissues and found MRI-based adrenal gland volume as a marker of unfavorable adipose tissue distribution, irrespective of anthropometric measurements and largely independent of cardiometabolic risk factors. Interestingly, our results showed that adrenal gland enlargement was particularly associated with increased VAT and hepatic fat whereas the associations with smaller adipose tissue depots such as pancreatic fat or epi- and pericardial fat were partly affected by cardiometabolic risk factors and seemed to be mainly mediated by VAT.

As an imaging biomarker, adrenal gland volume could therefore serve as an indication of metabolic changes and thus contribute to individual risk stratification through comprehensive whole-body MR-imaging in the framework of a future patient-adapted preventive medical setting.

### Supplementary Information

Supplementary Material 1: Supplementary Table 1. Results from a linear regression model with outcome adipose tissue and exposure left or right adrenal gland volume. Outcomes and exposures were standardized before analysis. Outcomes HFF, PDFF_pancreas_ and renal sinus fat were log-transformed before analysis and estimates represent percent change of the mean. For all other outcomes, estimates are given as beta coefficients. Adjustments: Model 1: age and sex, Model 2: age, sex and lifestyle factors (alcohol consumption, smoking, physical activity), Model 3: age, sex, lifestyle and metabolic risk factors (hypertension, diabetes, increased triglycerides), Model 4: age, sex, lifestyle, metabolic risk factors and BMI. Model 5: age, sex, lifestyle, metabolic risk factors and VAT.

Supplementary Material 2: Supplementary Figure 1. Correlation of adrenal gland volume with TAT, VAT and SAT.

Supplementary Material 3: Supplementary Figure 2. Correlation of adrenal gland volume with adipose tissue depots and metabolic risk factors in men.

Supplementary Material 4: Supplementary Figure 3. Correlation of adrenal gland volume with adipose tissue depots and metabolic risk factors in women. Estimate denotes Spearman’s correlation coefficient.

Supplementary Material 5: Supplementary Figure 4. Association of adrenal gland volume with adipose tissue compartments when adjusting for anthropometric factors. Results from a linear regression model with outcome adipose tissue and exposure adrenal gland volume. Outcomes and exposure were standardized before analysis. Outcomes HFF, PDFF_pancreas_ and renal sinus fat were log-transformed before analysis and estimates represent percent change of the mean. For all other outcomes, estimates are given as beta coefficients. Adjustments correspond to Model 3, i.e. age, sex, lifestyle (alcohol consumption, smoking, physical activity) and metabolic risk factors (hypertension, diabetes, increased trigylcerides) plus the anthropometric factor denoted in the first column.

Supplementary Material 6: Supplementary Figure 5. Association of adrenal gland volume with TAT in different subgroups. Results from a linear regression model with outcome TAT and exposure adrenal gland volume. Outcome and exposure were standardized before analysis. Estimates are given as beta coefficients Adjustments: age, sex, lifestyle and metabolic risk factors and BMI.

Supplementary Material 7: Supplementary Figure 6. Associations of adrenal gland volume with adipose tissue compartments in men.

Supplementary Material 8: Supplementary Figure 7. Associations of adrenal gland volume with adipose tissue compartments in women.

## Data Availability

The datasets used and analyzed during the current study are available from the corresponding author on reasonable request.

## Abbreviations

BMI: Body mass index
CI: Confidence interval
CT: Computed tomography
FOV: Field of view
FPG: Fasting plasma glucose
HDL: High density lipoprotein
HFF: Hepatic fat fraction
HPA: Hypothalamic-pituitary-adrenal (axis)
KORA: Cooperative Health Research in the Augsburg Region, Germany
LDL: Low density lipoprotein
MRI: Magnetic resonance imaging
oGTT: Oral glucose tolerance test
OR: Odds ratio
PDFF: Proton-density fat fraction
ROI: Region of interest
SAT: Subcutaneous adipose tissue
SD: Standard deviation
TAT: Total adipose tissue
TE: Echo time
TR: Repetition time
T2DM: Type 2 diabetes mellitus
VAT: Visceral adipose tissue
WHO: World Health Organization
