# Supplementary material for: Associations between adrenal gland volume and adipose tissue compartments – a whole body MRI study
Source: Nutr Metab (Lond). 2024 Jul 9;21:45. doi: 10.1186/s12986-024-00823-x (PMC11234623; doi:10.1186/s12986-024-00823-x)
Supplement: Supplementary file 1 — Supplementary Material 1: Supplementary Table 1. Results from a linear regression model with outcome adipose tissue and exposure left or right adrenal gland volume. Outcomes and exposures were standardized before analysis. Outcomes HFF, PDFFpancreas and renal sinus fat were log-transformed before analysis and estimates represent percent change of the mean. For all other outcomes, estimates are given as beta coefficients. Adjustments: Model 1: age and sex, Model 2: age, sex and lifestyle factors (alcohol consumption, smoking, physical activity), Model 3: age, sex, lifestyle and metabolic risk factors (hypertension, diabetes, increased triglycerides), Model 4: age, sex, lifestyle, metabolic risk factors and BMI. Model 5: age, sex, lifestyle, metabolic risk factors and VAT. [file 12986_2024_823_MOESM1_ESM.pdf]

		Model 1			Model 2			Model 3			Model 4			Model 5		
		Est	95%-CI	p-value		95%-CI	p-value		95%-CI	p-value		95%-CI	p-value		95%-CI	p-value
TAT	left	0.66	[0.56, 0.77]	1.83E-28	0.63	[0.52, 0.73]	6.37E-26	0.51	[0.40, 0.62]	6.66E-19	0.16	[0.10, 0.23]	2.13E-06	0.1	[0.01, 0.18]	0.023
	right	0.62	[0.51, 0.73]	4.71E-24	0.58	[0.47, 0.69]	1.34E-21	0.45	[0.33, 0.56]	2.56E-13	0.14	[0.07, 0.21]	6.37E-05	0.12	[0.04, 0.20]	0.002
VAT	left	0.58	[0.49, 0.66]	1.99E-32	0.55	[0.46, 0.64]	3.79E-29	0.44	[0.36, 0.52]	1.36E-21	0.29	[0.20, 0.37]	4.13E-11			
	right	0.51	[0.42, 0.60]	2.70E-24	0.48	[0.39, 0.57]	1.87E-21	0.34	[0.25, 0.44]	5.91E-12	0.19	[0.10, 0.28]	2.33E-05			
SAT	left	0.54	[0.43, 0.65]	8.98E-19	0.51	[0.40, 0.62]	4.43E-17	0.42	[0.30, 0.53]	5.43E-12	0.03	[-0.04, 0.09]	4.47E-01	0.14	[0.02, 0.26]	0.023
	right	0.52	[0.41, 0.63]	2.09E-17	0.49	[0.38, 0.60]	1.09E-15	0.4	[0.28, 0.52]	4.07E-10	0.06	[-0.00, 0.13]	6.24E-02	0.18	[0.06, 0.29]	0.002
HFF	left	1.68	[1.51, 1.86]	1.99E-19	1.67	[1.49, 1.86]	2.59E-18	1.45	[1.31, 1.6]	8.57E-12	1.3	[1.16, 1.45]	3.29E-06	1.14	[1.02, 1.27]	0.019
	right	1.63	[1.46, 1.82]	3.09E-17	1.62	[1.45, 1.8]	5.83E-16	1.36	[1.21, 1.52]	1.25E-07	1.22	[1.08, 1.36]	7.34E-04	1.11	[1.00, 1.23]	0.051
PDFF_pancreas	left	1.42	[1.27, 1.6]	9.93E-09	1.4	[1.25, 1.58]	7.49E-08	1.36	[1.2, 1.55]	2.90E-06	1.23	[1.07, 1.42]	3.20E-03	1.07	[0.93, 1.25]	0.301
	right	1.38	[1.22, 1.55]	2.46E-07	1.36	[1.21, 1.54]	1.37E-06	1.32	[1.15, 1.51]	8.56E-05	1.2	[1.03, 1.38]	1.47E-02	1.09	[0.95, 1.25]	0.211
Renal Sinus fat	left	1.26	[1.12, 1.42]	1.10E-04	1.23	[1.09, 1.39]	6.00E-04	1.2	[1.05, 1.35]	6.38E-03	1.15	[1.00, 1.32]	4.64E-02	1.07	[0.93, 1.25]	0.359
	right	1.15	[1.02, 1.3]	1.83E-02	1.13	[1, 1.27]	5.62E-02	1.08	[0.95, 1.23]	2.39E-01	1.03	[0.9, 1.19]	6.56E-01	0.98	[0.95, 1.25]	0.759
Epicardial fat	left	0.35	[0.24, 0.46]	2.87E-09	0.35	[0.23, 0.46]	1.11E-08	0.33	[0.20, 0.45]	3.51E-07	0.24	[0.10, 0.37]	5.57E-04	0.04	[-0.09, 0.17]	0.555
	right	0.24	[0.13, 0.36]	5.74E-05	0.23	[0.11, 0.35]	1.62E-04	0.18	[0.05, 0.31]	7.80E-03	0.08	[-0.06, 0.21]	2.73E-01	-0.06	[-0.19, 0.06]	0.341
Pericardial fat	left	0.38	[0.28, 0.48]	6.38E-13	0.37	[0.27, 0.47]	8.96E-12	0.33	[0.22, 0.44]	6.26E-09	0.24	[0.12, 0.35]	8.40E-05	-0.01	[-0.11, 0.10]	0.918

	right	0.32	[0.22, 0.42]	3.02E-09	0.3	[0.20, 0.41]	4.25E-08	0.23	[0.12, 0.35]	1.03E-04	0.13	[0.01, 0.25]	2.98E-02	-0.03	[-0.13, 0.07]	0.536
