# Supplementary figures and images for: Associations between adrenal gland volume and adipose tissue compartments – a whole body MRI study
Source: Nutr Metab (Lond). 2024 Jul 9;21:45. doi: 10.1186/s12986-024-00823-x (PMC11234623; doi:10.1186/s12986-024-00823-x)

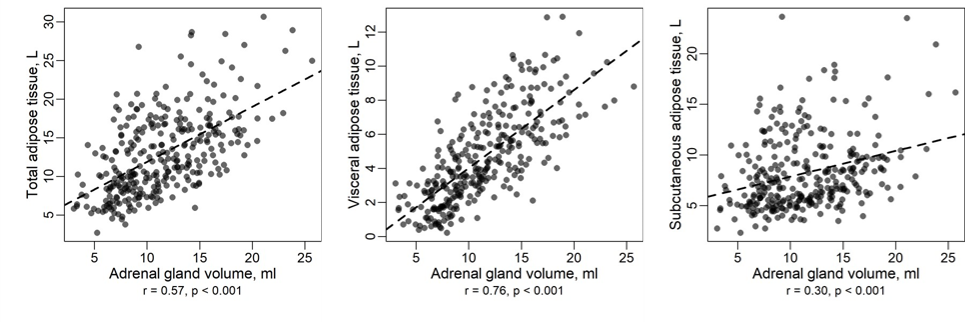

Supplement: Supplementary file 2 — Supplementary Material 2: Supplementary Figure 1. Correlation of adrenal gland volume with TAT, VAT and SAT. [file 12986_2024_823_MOESM2_ESM.png]

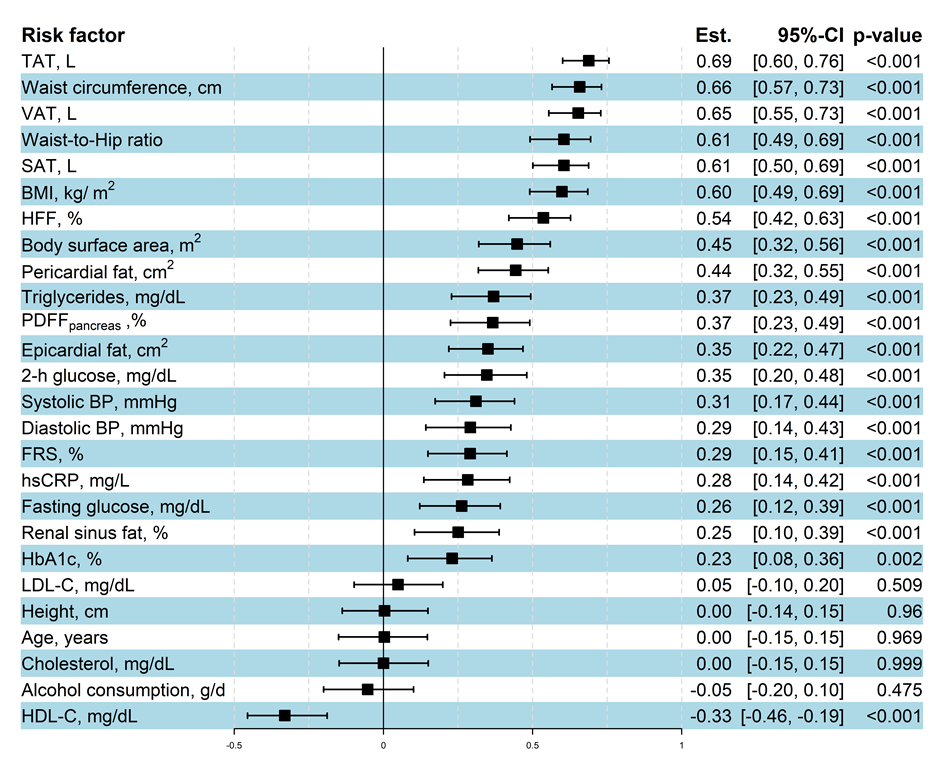

Supplement: Supplementary file 3 — Supplementary Material 3: Supplementary Figure 2. Correlation of adrenal gland volume with adipose tissue depots and metabolic risk factors in men. [file 12986_2024_823_MOESM3_ESM.png]

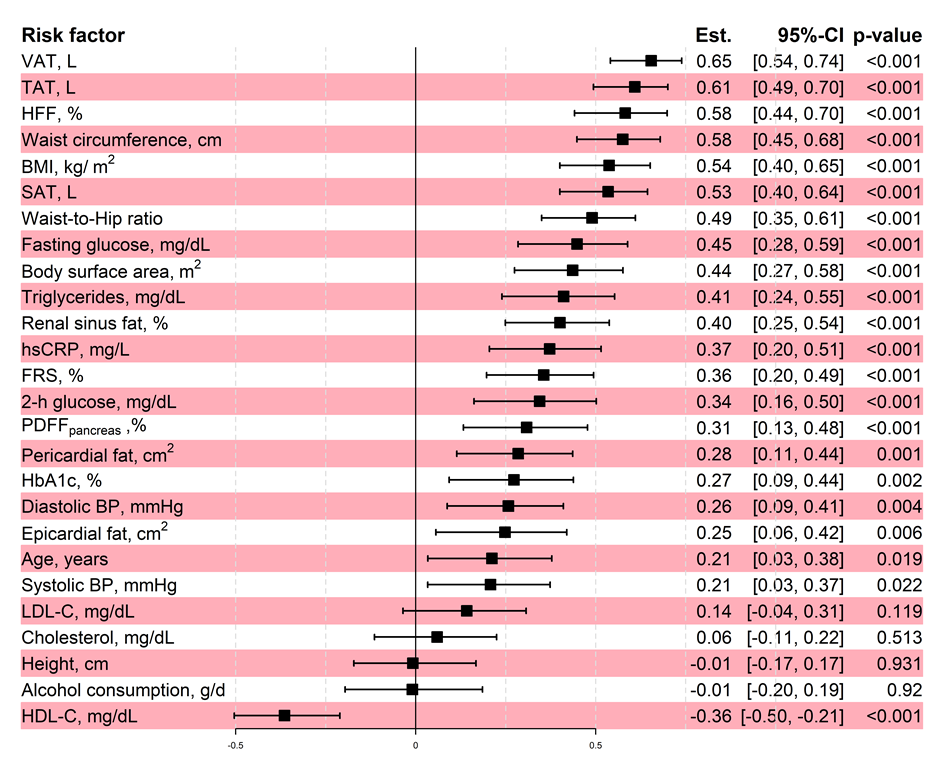

Supplement: Supplementary file 4 — Supplementary Material 4: Supplementary Figure 3. Correlation of adrenal gland volume with adipose tissue depots and metabolic risk factors in women. Estimate denotes Spearman’s correlation coefficient. [file 12986_2024_823_MOESM4_ESM.png]

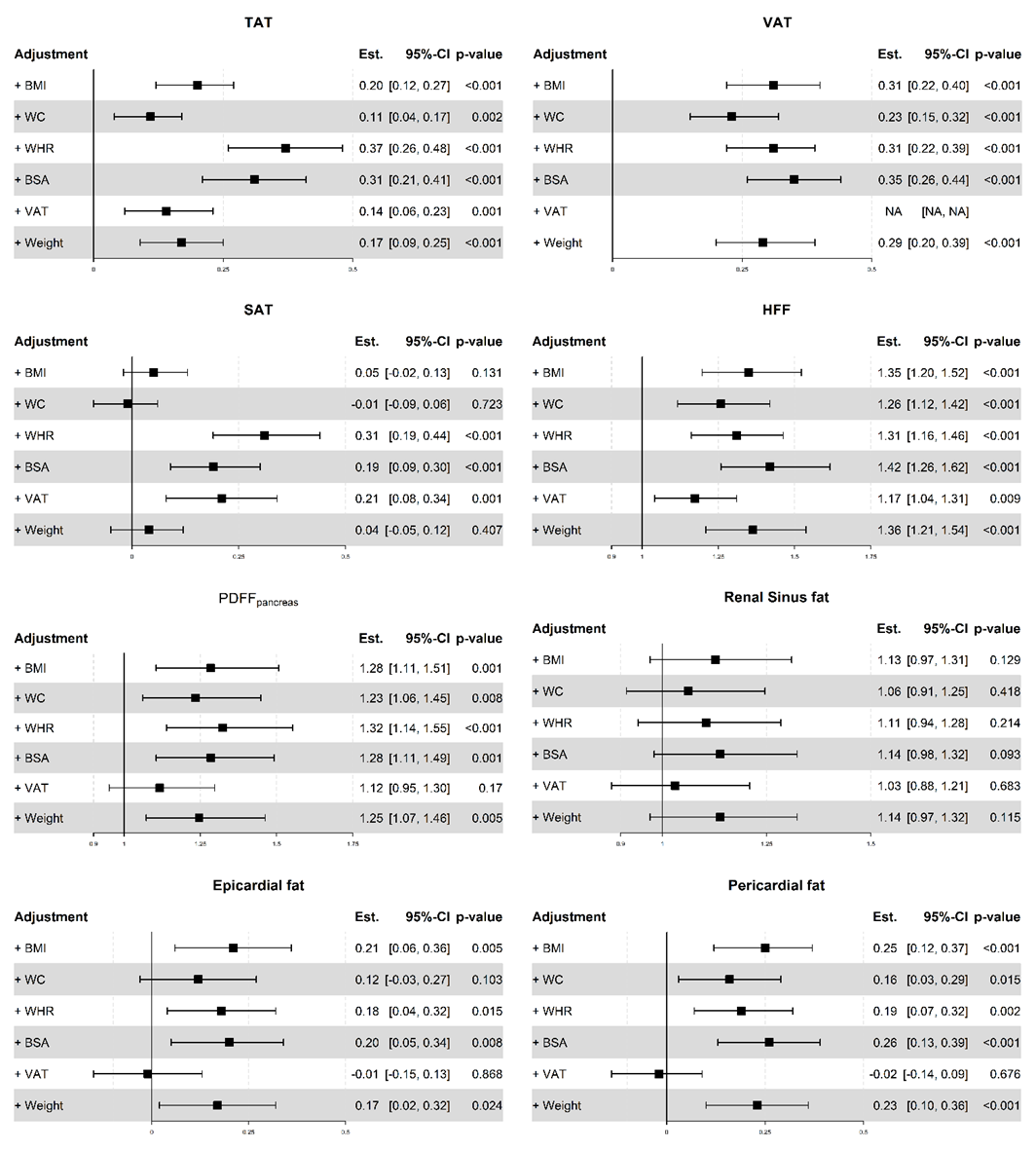

Supplement: Supplementary file 5 — Supplementary Material 5: Supplementary Figure 4. Association of adrenal gland volume with adipose tissue compartments when adjusting for anthropometric factors. Results from a linear regression model with outcome adipose tissue and exposure adrenal gland volume. Outcomes and exposure were standardized before analysis. Outcomes HFF, PDFFpancreas and renal sinus fat were log-transformed before analysis and estimates represent percent change of the mean. For all other outcomes, estimates are given as beta coefficients. Adjustments correspond to Model 3, i.e. age, sex, lifestyle (alcohol consumption, smoking, physical activity) and metabolic risk factors (hypertension, diabetes, increased trigylcerides) plus the anthropometric factor denoted in the first column. [file 12986_2024_823_MOESM5_ESM.png]

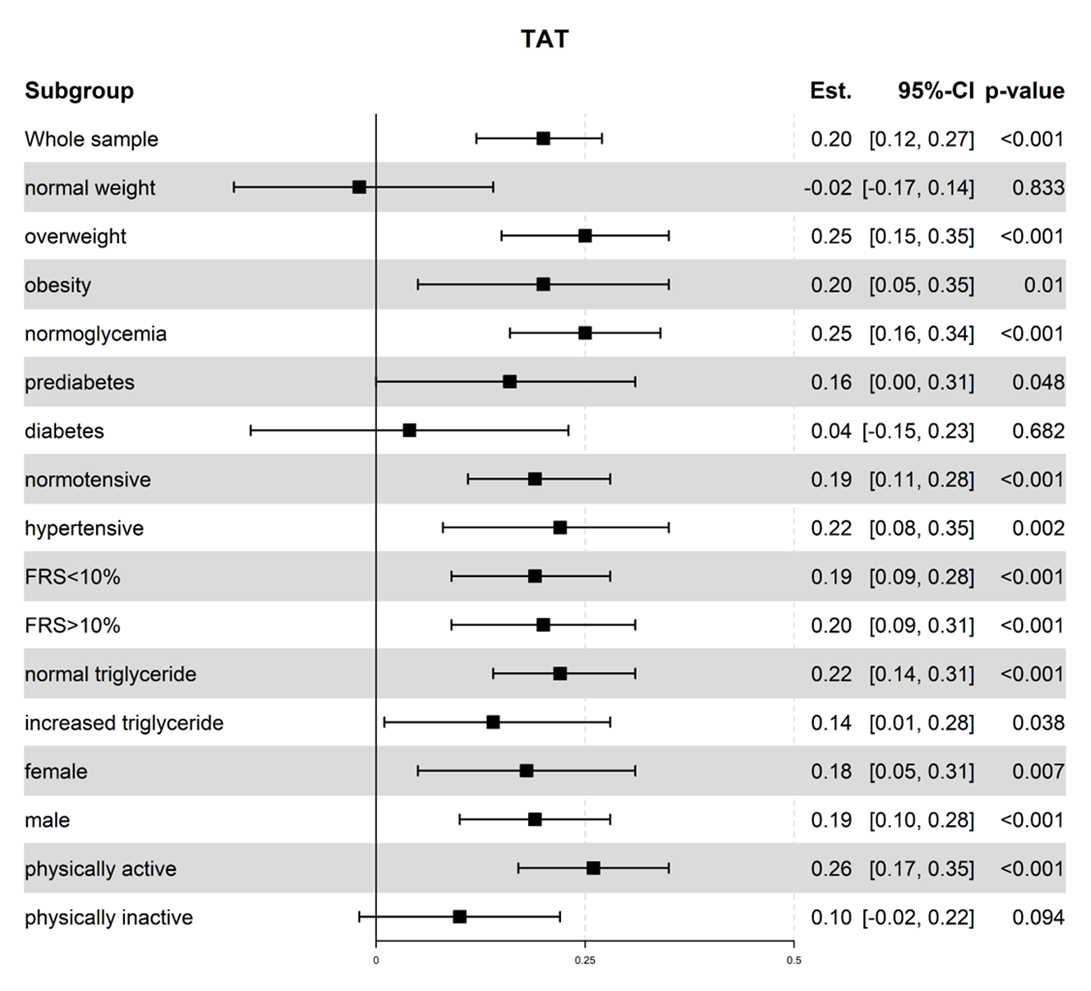

Supplement: Supplementary file 6 — Supplementary Material 6: Supplementary Figure 5. Association of adrenal gland volume with TAT in different subgroups. Results from a linear regression model with outcome TAT and exposure adrenal gland volume. Outcome and exposure were standardized before analysis. Estimates are given as beta coefficients Adjustments: age, sex, lifestyle and metabolic risk factors and BMI. [file 12986_2024_823_MOESM6_ESM.png]

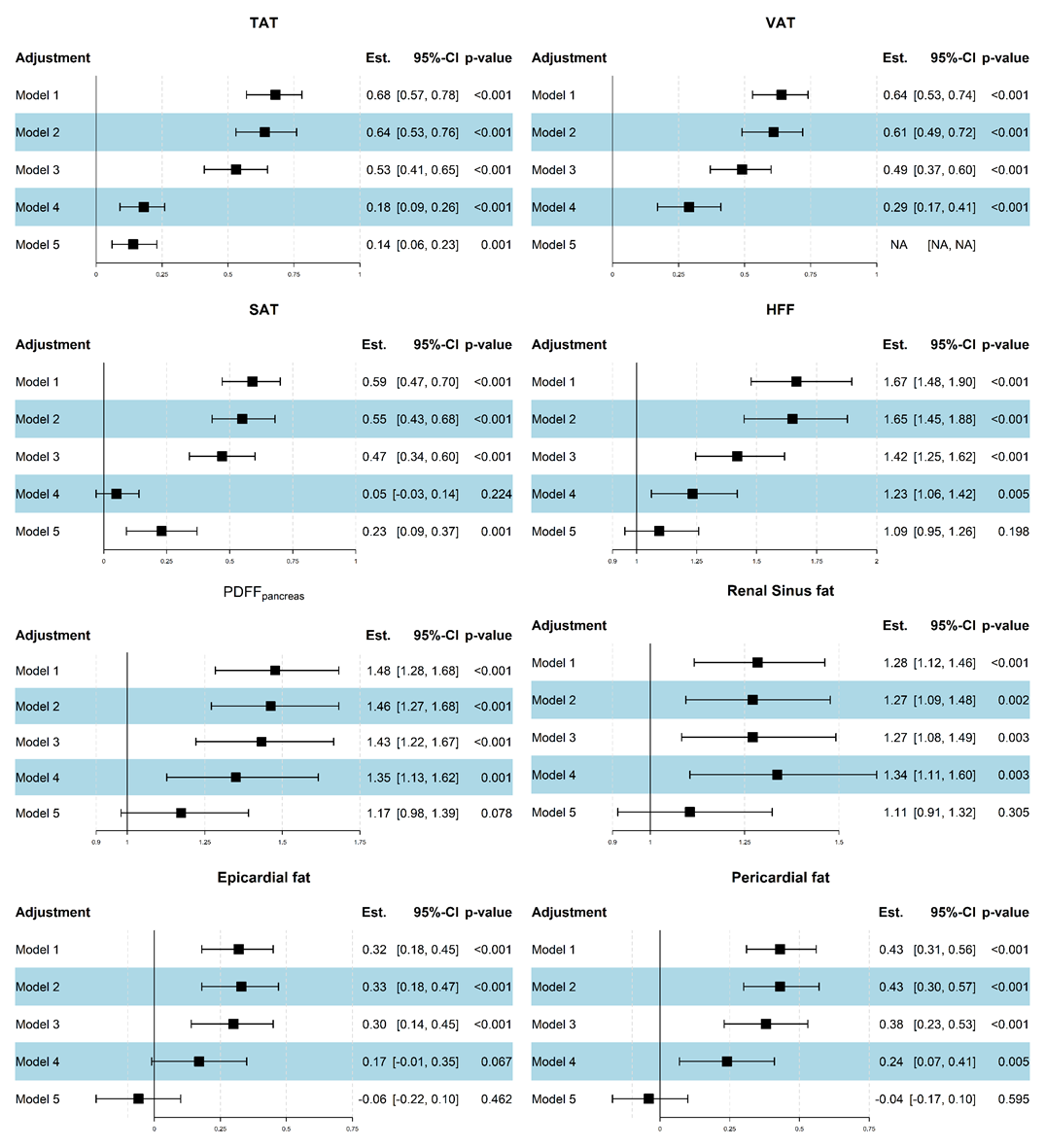

Supplement: Supplementary file 7 — Supplementary Material 7: Supplementary Figure 6. Associations of adrenal gland volume with adipose tissue compartments in men. [file 12986_2024_823_MOESM7_ESM.png]

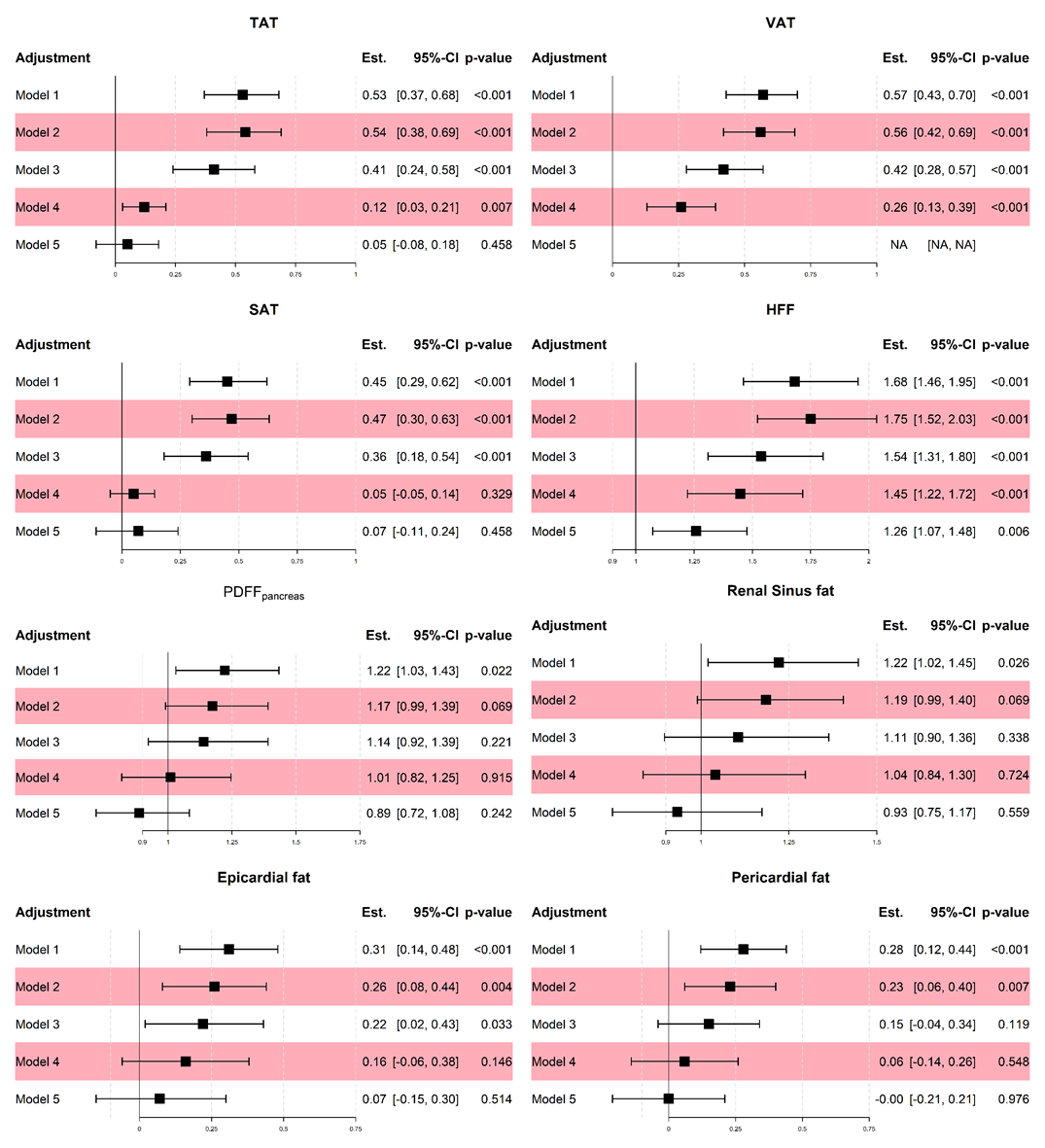

Supplement: Supplementary file 8 — Supplementary Material 8: Supplementary Figure 7. Associations of adrenal gland volume with adipose tissue compartments in women. [file 12986_2024_823_MOESM8_ESM.png]
